# Smartphone-Enabled Health Coach Intervention for People With Diabetes From a Modest Socioeconomic Strata Community: Single-Arm Longitudinal Feasibility Study

**DOI:** 10.2196/jmir.3180

**Published:** 2014-06-06

**Authors:** Noah Wayne, Paul Ritvo

**Affiliations:** ^1^School of Kinesiology & Health ScienceFaculty of HealthYork UniversityToronto, ONCanada; ^2^Department of PsychologyFaculty of HealthYork UniversityToronto, ONCanada; ^3^ResearchPrevention and Cancer ControlCancer Care OntarioToronto, ONCanada; ^4^Department of PsychiatryFaculty of MedicineUniversity of TorontoToronto, ONCanada; ^5^Department of Family and Community MedicineFaculty of MedicineUniversity of TorontoToronto, ONCanada; ^6^Division of Epidemiology and BiostatisticsOntario Cancer InstitutePrincess Margaret HospitalToronto, ONCanada

**Keywords:** diabetes mellitus, type 2, health coaching, telehealth

## Abstract

**Background:**

Lower socioeconomic strata (SES) populations have higher chronic disease risks. Smartphone-based interventions can support adoption of health behaviors that may, in turn, reduce the risks of type 2 diabetes-related complications, overcoming the obstacles that some patients may have with regular clinical contact (eg, shiftwork, travel difficulties, miscommunication).

**Objective:**

The intent of the study was to develop and test a smartphone-assisted intervention that improves behavioral management of type 2 diabetes in an ethnically diverse, lower SES population within an urban community health setting.

**Methods:**

This single-arm pilot study assessed a smartphone application developed with investigator assistance and delivered by health coaches. Participants were recruited from the Black Creek Community Health Centre in Toronto and had minimal prior experience with smartphones.

**Results:**

A total of 21 subjects consented and 19 participants completed the 6-month trial; 12 had baseline glycosylated hemoglobin (HbA1c) levels >7.0% and these subjects demonstrated a mean reduction of 0.43% (SD 0.63) (*P*<.05) with minimal changes in medication.

**Conclusions:**

This project supported the feasibility of smartphone-based health coaching for individuals from lower SES with minimal prior smartphone experience.

##  Introduction

### Background

A consensus of medical professionals and academic researchers indicates that type 2 diabetes mellitus (T2DM) is a chronic condition that progresses to more debilitating complications if certain unhealthy behaviors persist [1]. Regular exercise conversely prevents deteriorating health and disease onset [2] and has measurable benefits for T2DM-diagnosed populations [3,4]. Because high carbohydrate diets increase risks for diabetes-related complications due to chronic hyperglycemia, dietary modification can also result in risk reductions [5]. The adoption of optimal health behaviors in those diagnosed with T2DM requires behavior change and support for diabetic individuals from lower socioeconomic strata (SES) is especially important as this population confronts additional challenges in maintaining good health [6]. Data from the Canadian Healthy Community Survey (2005) suggest individuals from the lowest income group are over four times more likely to have T2DM [7]. Furthermore, education and personal wealth variables, typically viewed as SES proxies, are the strongest predictors of premature death associated with T2DM [8]. Despite recent surges of interest in disease incidence related to SES, little attention has been paid to urban, low SES immigrant/minority groups. As our experience indicates, these individuals are often less willing to volunteer for research and are less reliable subjects after enrollment. This is mainly related to the competing demands they confront and the lack of flexibility in their working conditions.

Health coaches promote adoption and maintenance of health behaviors, using validated theoretical frameworks (eg, motivational interviewing [9] and cognitive behavioral therapy [10]). Health coaches primarily focus on helping patients define and attain personal goals and discover intrinsic health-oriented motivations [11]. Recent trials involving health coaching in chronic disease demonstrate positive gains for patients, such as increased exercise and medication adherence [11,12], improved psychological functioning [11], and more positive illness-coping strategies [11].

Mobile technologies complement health coaching by enabling patients and coaches to maintain multiple channels of contact via remote monitoring, voice, and text message communications. The use of mobile phones potentially provides unprecedented precision in supporting health-related behavior since it facilitates responses to immediate needs and serves to maintain communication consistency. Once an individual decides on the intensity, frequency, and duration of contacts with the health coach, it is possible to detect non-adherence lapses quickly to the point where supportive-corrective responses can be provided while the non-adherent pattern is still unfolding. Reminder and reinforcement messages of different types can be sent to patients at any hour of day or evening, enabling interactions that purposefully blend with the patient’s daily lifestyle.

Remote monitoring has been associated in numerous controlled studies with significant benefits in improving blood pressure and blood glucose regulation [13-16], exercise adherence [17], and dietary control [18,19]. Mobile technologies enable immediate and inexpensive communication with patients, exemplified in the use of text messages (SMS) to boost medication adherence and decrease viral load in HIV-positive Kenyan populations [20], and to deliver supportive SMS to patients at risk for developing type 2 diabetes [21], and have demonstrated results with a variety of other chronic medical conditions [22].

### NexJ Connected Health and Wellness Platform (CHWP)

The Connected Health and Wellness Platform (CHWP) Health Coach app is designed to support multi-channel communications between clients and health coaches and supportive family members. The app was collaboratively designed by software developers (NexJ Systems Inc.) and study investigators to support participants in electronically tracking health behaviors (eg, exercise, diet, stress reduction practices) and self-monitoring health data (eg, blood glucose, blood pressure, mood, pain, energy). Provider-client communications require two-way, certificate-based authentication and passwords stored in encrypted columns, with entered data recalled by client and health coach through a secure online portal.

##  Methods

### Study Design

This experimental pre/post, single-arm trial assessed a 24-week intervention where interactions in person, by phone, and by smartphone (eg, secure messaging, email) with a personal health coach supported adoption of and adherence to self-generated health-behavior change goals. The primary study outcome was glycosylated hemoglobin (HbA1c) assessed at baseline and 24 weeks. HbA1c is a clinical indicator of glucose regulation correlated with debilitating and costly diabetic complications. The clinical goal for self-management of diabetes is an HbA1c of 7.0% or less, although further reductions are preferred. Interventions that reduce HbA1c in elevated risk populations are of significant value in diabetes care.

### Health Coaching Intervention

The health coach intervention was carried out by a graduate student trained in behavior change techniques. After obtaining informed consent and collecting demographic information, baseline lab reports, and psychometric measures, the participants and the health coach communicated about eating, physical activity patterns, and overall health goals. Wellness plans were collaboratively created in multiple interactions focused on exercise instruction and reviews of electronic monitoring entries, with diet and medication guidelines set by primary care physicians and dieticians.

### Recruitment

Participants were recruited at the Black Creek Community Health Centre in Toronto, Ontario, Canada. Recruitment was through health care provider referral and poster advertising. Eligible participants were patients over 18 years old, diagnosed with type 2 diabetes, and able to read and speak English. Participants were excluded if their baseline HbA1c was greater than 9.5%. All study procedures were approved by the York University Human Participants Research Committee and participants signed an informed consent.

### NexJ Health Coach App Access

All clients were given access to the custom smartphone app, Health Coach, on a loaned Blackberry Curve 8900 with full data access for the duration of the trial (n=19), unless they possessed a smartphone (n=2).

### App Feedback and Development

**Figure 1:**
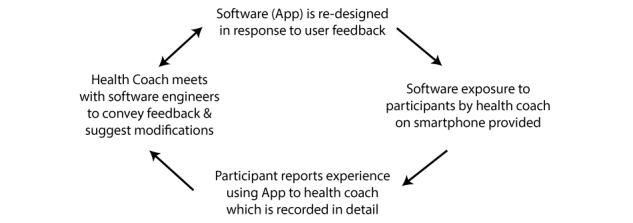
Software improvement cycle. Feedback loop conveys user experience and smartphone software redesign.

Research staff collected participant experience with version 1.0 of the Health Coach app, reporting errors and overall feedback. Feedback was organized and relayed back to the software design team as described in Figure 1.

**Figure 2:**
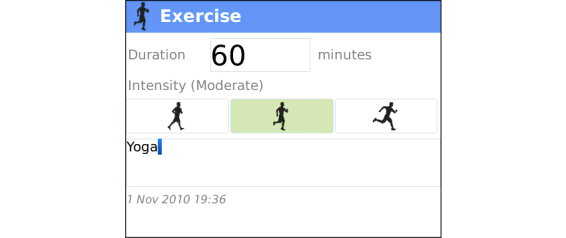
Exercise Tracker is designed to easily track multiple exercise modalities. Users can log duration of exercise, rate perceived intensity (light, moderate, vigorous), and enter additional text comments.

**Figure 3:**
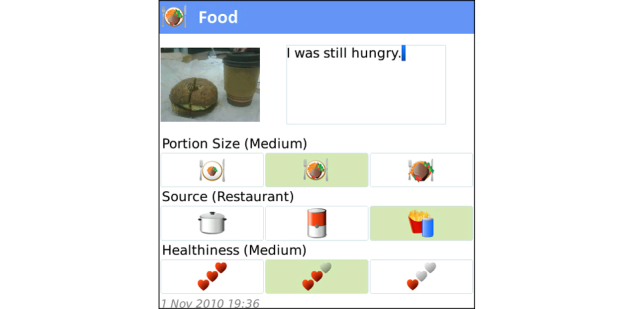
Food Tracker automatically triggers the smartphone’s camera, enabling photo capture of meals. Users can subjectively rate food portion (small, moderate, large), source (home-made, packaged, restaurant served), and healthiness (not so healthy, moderately healthy, very healthy).

**Figure 4:**
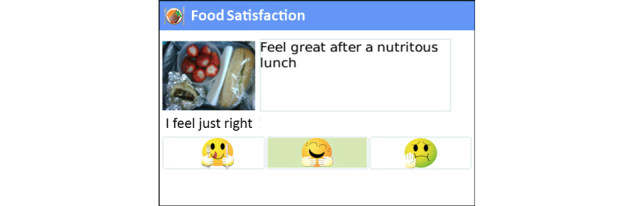
Satisfaction survey: at a customizable timeframe (usually 20 minutes), the program prompts for reports on satiety level (not enough, just right, too full).

**Figure 5:**
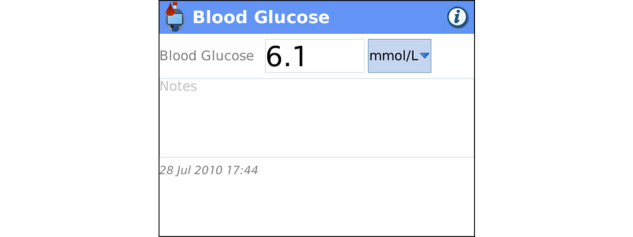
Blood Glucose Tracker: Clients enter blood glucose level and comments on readings.

**Figure 6:**
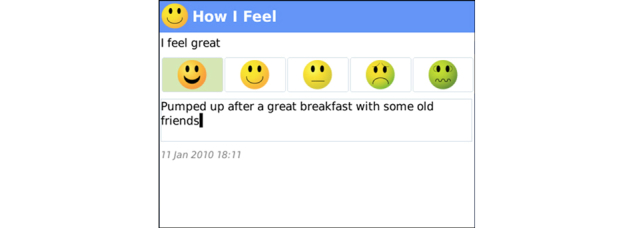
Mood Tracker: Clients enter “How They Feel” using a simple 5-pt scale: I feel (great, very good, good, bad, very bad) and comment on entry, which is time-stamped.

**Figure 7:**
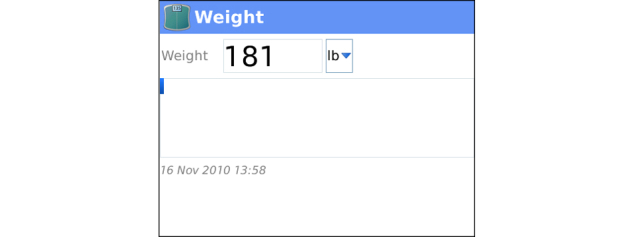
Weight Tracker: Clients enter weight and enter comments on the reading.

**Figure 8:**
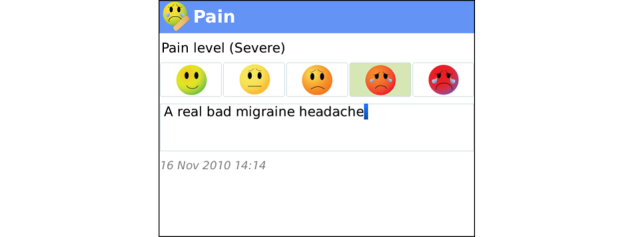
Pain Tracker: Clients can enter subjective pain ratings using a 5-pt scale: pain level is (none, mild, moderate, severe, very severe).

**Figure 9:**
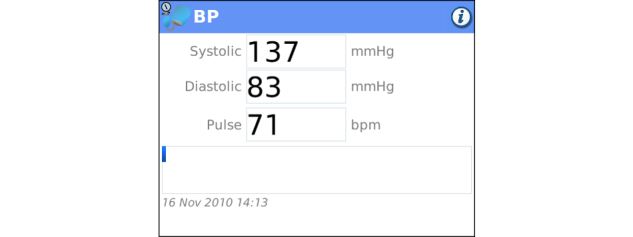
Blood Pressure Tracker: Clients enter blood pressure including systolic, diastolic, and heart rate and are able to comment on the reading.

**Figure 10:**
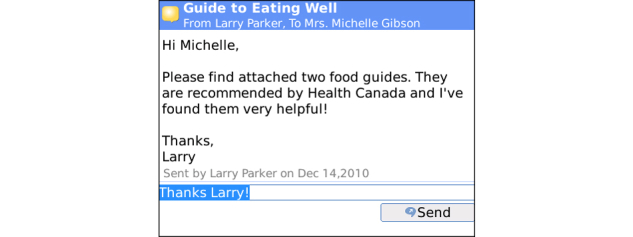
Messaging allows for two-way secure messaging between participant and health coach who can selectively promote healthy choices at pivotal times of client decision-making, providing support immediately after healthy behaviors have been logged, and/or addressing questions and/or sending relevant materials.

**Figure 11:**
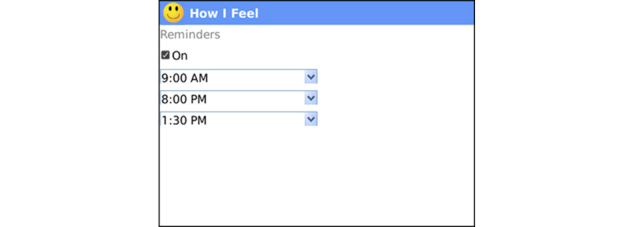
Reminders: The trackers use employ alarm-type entry reminders, which provide convenient ways to prompt clients to engage in health behaviors like exercise, dietary modifications, stress reduction, and self-reported mood. Reminders can be turned on and off easily by health coach and/or participant.

As the Health Coach app was version 1.0, periodic malfunctions hindered client communications during the trial. Due to the close relationship between the health coach and software production team, the feedback and user experience was communicated as received, resulting in upgrades installed on the server at frequent intervals. This feedback loop led to significant improvements in the software throughout the trial. Some of the most important modifications included user-interface enhancements, general usability, and solution of software instability issues. Screenshots of the mobile phone app with an explanation of the various trackers and functions are found in Figures 2-11.

### Statistical Analysis

Data was analyzed using SPSS (version 21.0, 2012, IBM, Chicago, IL). Descriptive statistics are reported (means and standard deviations). Differences in outcome variables (baseline to 24 weeks) were analyzed using a paired samples *t* test. Participants were split into groupings according to baseline assessments (HbA1c≥7.0% and HbA1c<7.0%). Significance was set to *P*<.05.

## Results

Of the 21 participants, final outcome variables were collected for 19. The primary reason for missing data was primary care physician failure to forward lab results (n=2).

Demographics are summarized in Table 1. There was a mean reduction of 0.28% (SD 0.57) (*P*=.05) found over the entire sample. Since participant glucose control varied across optimal levels at baseline, data was re-analyzed for those who began the trial with sub-optimally managed glucose and those with optimally managed glucose. A total of 12 participants started the trial with sub-optimally managed glycemic control (HbA1c≥7.0% [Diabetes Control and Complications Trial/DCCT method] or 53 mmol/mol [International Federation of Clinical Chemistry/IFCC method]) and experienced a greater mean reduction of 0.43% (SD 0.63) (*P*=.04) (see Table 2).

**Table 1:** Demographic characteristics at baseline (n=21).

Characteristic		n (%)
Age (years), mean (SD)		55.6 (12.3)
**Sex**		
	Male	9 (43%)
	Female	12 (57%)
**Marital status**		
	Single	5 (24%)
	Married or common law	14 (67%)
	Widowed	2 (10%)
**Children**		
	Yes	18 (86%)
	No	3 (14%)
**Educational background**		
	Less than high school	3 (14%)
	Completed high school	4 (19%)
	Some college/university	7 (33%)
	College diploma	6 (29%)
	University degree	1 (5%)
**Employment**		
	Full-time	12 (57%)
	Part-time	2 (10%)
	Not presently employed	7 (33%)
**Ethno-cultural group**		
	Hispanic	3 (14%)
	African	3 (14%)
	Caribbean	3 (14%)
	South Asian	3 (14%)
	Caucasian	9 (43%)

**Table 2:** Change in outcomes of patients participating in the Health Coach intervention.

Outcome		n	Baseline, mean (SD)	Post, mean (SD)	Mean change, mean (SD)	*P* value
**Entire sample**						
	HbA1c^a^ (%)	19	7.58 (1.13)	7.31 (0.95)	−0.28 (0.57)	.05
	Weight (kg)	14	94.6 (16.8)	93.2 (15.8)	−1.3 (1.9)	.02
	BMI^b^	13	34.4 (5.5)	33.9 (5.3)	−0.4 (0.7)	.05
	Waist circumference (cm)	11	109.4 (16.1)	112.1 (16.1)	2.7 (4.3)	.06
**Baseline A1c≥7.0%**						
	HbA1c (%)	12	8.26 (0.80)	7.83 (0.78)	−0.43 (0.63)	.04
	Weight (kg)	9	100.1 (18.0)	98.1 (17.1)	−1.9 (1.7)	.01
	BMI	8	36.2 (5.8)	35.6 (5.7)	−0.7 (0.7)	.37
	Waist circumference (cm)	7	114.4 (17.1)	116.5 (16.4)	2.1 (5.3)	.33
**Baseline A1c<7.0%**						
	HbA1c (%)	7	6.43 (0.39)	6.41 (0.38)	−0.01 (0.32)	.91
	Weight (kg)	5	84.6 (8.7)	84.4 (8.8)	−0.2 (1.8)	.81
	BMI	5	31.4 (3.7)	31.3 (3.8)	−0.1 (0.7)	.80
	Waist circumference (cm)	4	100.6 (11.0)	104.4 (10.0)	3.8 (1.6)	.02

^a^HbA1c: hemoglobin A1c (glycosylated hemoglobin)

^b^BMI: body mass index

##  Discussion

### Principal Results

In this trial, patients with a range of glucose regulation efficacy were recruited to pilot a smartphone-based mobile software app and personal health coach program. Given the objective of demonstrating intervention efficacy in poorly managed diabetic clients, our analysis focused on subjects with a baseline HbA1c>7.0% (53 mmol/mol). These participants, who started at a poorly managed level, showed a mean reduction of 0.43% (SD 0.63) (*P*=.04), demonstrating the potential clinical relevance of the intervention.

### Socioeconomic Strata and Intervention Applicability

Lower SES populations often have difficulty navigating and accessing the health care system [23] to a degree where SES appears to be the best predictor of health status in Canada and the United States [24,25], with SES-related factors manifesting as substantial barriers to the health of many Canadians. This intervention attempted to address some of these issues by engaging participants in a health coaching relationship to overcome accessibility barriers. During the course of the intervention, it was observed that participants were sometimes prevented from attending appointments with their health care team due to familial obligations and work obligations (mainly shift work). With low workplace flexibility, when work had to be interrupted to attend a health care session, losing out on the day’s pay was a significant obstacle. The intervention reduced this barrier by providing 24-hour electronic access to the health coach, enabling participants to initiate communication when possible and convenient.

Of our study sample, 34% completed either a college or university degree, compared to the 59% of Ontario’s population (and 53% of Canada’s population) who have a university or college level designation [26]. Education is a commonly used proxy of socioeconomic strata, but educated immigrants to Canada are frequently unable to work in their former disciplines at their achieved educational levels due to domestic policies [27]. The intervention demonstrated the effectiveness of a personalized, electronically assisted health coaching intervention in an underserved population that is not typically the focus of technology-assisted health research. Most participants (n=19) did not own a smartphone and were loaned a device for the trial duration. Nonetheless, as the costs of mobile technology decrease, mobile technology interventions will be increasingly feasible and useful at all SES levels.

### From Single-Arm Pilot to Randomized Controlled Trial

The pilot study was intended to generate results guiding the eventual design of a randomized controlled trial (RCT). Several points of guidance were readily apparent. First, the lower SES participants, according to our pilot experience, would not likely sustain participation if they perceived that randomization to the control group resulted in an inferior intervention. This was due to generic participation obstacles, especially taking time out of inflexible work schedules to attend assessment sessions. This observation combined with our interest in seeing what additional benefits were attributable to health coaching with the smartphone software vs health coaching alone. Accordingly, the health coaching intervention was designed to be fundamentally equivalent across comparison arms except for use of the smartphone plus software in the experimental group. Second, our experience with primary care providers involved their inconsistent provision of HbA1c tests. Accordingly, we have ensured a point-of-care HbA1c Analyzer is available (via finger-prick A1c blood samples) throughout the current RCT.

### Limitations

This pilot study enrolled a small convenience sample with no control group, limiting the generalizability of intervention results. Throughout the pilot trial, temporary software malfunctions and upgrades inevitably resulted in service disruptions. Although participants could directly log healthy behaviors via smartphone, their self-report could be falsified or exaggerated. Future studies can employ Bluetooth connected technology (ie, glucometers, accelerometers) to omit some self-report biases. To more rigorously assess intervention efficacy, the RCT now in the field is being undertaken with stabilized, consistently functional software. The goal is to assess whether health coaching without vs with the use of the smartphone software is equivalent (or non-inferior). In order to address this question, subjects were randomly allocated to experimental and control groups and the same coaches delivered health coaching in both arms. This approach aims to better understand which intervention features are most important to effective intervention. We understand that there are limitations to this assessment approach but it represents an important step in investigating these interventions.

### Comparison With Prior Work

The most comparable intervention is the WellDoc diabetes trial [28-30] in which 26 primary health practices were randomized to provide one of four possible health coach intervention options to their patients. Across participating practices, 163 patients were intervened with intensities ranging from usual care to use of smartphone-assisted health coaching. Investigators found significant decreases in HbA1c in the highest intensity group. In that trial, participants on Medicaid and Medicare and those without health insurance were excluded. Our trial specifically targets individuals from a lower-resource sector of a large Canadian city, most of whom would have been excluded from the WellDoc trial. Since the association between type 2 diabetes and poverty has been well demonstrated [6,7,8], our interests focus on interventions that serve people of all SES and have demonstrated efficacy with subjects from lower SES.

### Conclusions

As mobile technology becomes more accessible, electronically assisted health coaching may emerge as a viable and effective means of managing chronic conditions through improved health behaviors across all SES. To help understand what parts of the intervention were responsible for changes in behavior (health coaching, remote monitoring), the RCT currently being conducted will assess the effectiveness of health coaching in type 2 diabetic patients both with and without the use of smartphone technology at multiple sites with diverse populations.

## Abbreviations

BMI: body mass index
HbA1c: hemoglobin A1c (glycosylated hemoglobin)
RCT: randomized controlled trial
SES: socioeconomic strata
T2DM: type 2 diabetes mellitus

## References

[1] Canadian Diabetes Association Clinical Practice Guidelines Expert Committee (2013). Can J Diabetes.

[2] Burr JF, Rowan CP, Jamnik VK, Riddell MC (2010). The role of physical activity in type 2 diabetes prevention: physiological and practical perspectives. Phys Sportsmed.

[3] Reid RD, Tulloch HE, Sigal RJ, Kenny GP, Fortier M, McDonnell L, Wells GA, Boulé NG, Phillips P, Coyle D (2010). Effects of aerobic exercise, resistance exercise or both, on patient-reported health status and well-being in type 2 diabetes mellitus: a randomised trial. Diabetologia.

[4] Church TS, Blair SN, Cocreham S, Johannsen N, Johnson W, Kramer K, Mikus CR, Myers V, Nauta M, Rodarte RQ, Sparks L, Thompson A, Earnest CP (2010). Effects of aerobic and resistance training on hemoglobin A1c levels in patients with type 2 diabetes: a randomized controlled trial. JAMA.

[5] Abete I, Astrup A, Martínez JA, Thorsdottir I, Zulet MA (2010). Obesity and the metabolic syndrome: role of different dietary macronutrient distribution patterns and specific nutritional components on weight loss and maintenance. Nutr Rev.

[6] Beryl Pilkington F, Daiski I, Bryant T, Dinca-panaitescu M, Dinca-panaitescu S, Raphael D (2010). The experience of living with diabetes for low-income Canadians. Canadian Journal of Diabetes.

[7] Dinca-Panaitescu S, Dinca-Panaitescu M, Bryant T, Daiski I, Pilkington B, Raphael D (2011). Diabetes prevalence and income: Results of the Canadian Community Health Survey. Health Policy.

[8] Saydah SH, Imperatore G, Beckles GL (2013). Socioeconomic status and mortality: contribution of health care access and psychological distress among U.S. adults with diagnosed diabetes. Diabetes Care.

[9] Miller WR, Rollnick S (1991). Motivational interviewing: preparing people to change addictive behavior.

[10] Sheldon B (2011). Cognitive-behavioural therapy: Research and practice in health and social care.

[11] Kreitzer MJ, Sierpina VS, Lawson K (2008). Health coaching: innovative education and clinical programs emerging. Explore (NY).

[12] Wolever RQ, Dreusicke M, Fikkan J, Hawkins TV, Yeung S, Wakefield J, Duda L, Flowers P, Cook C, Skinner E (2010). Integrative health coaching for patients with type 2 diabetes: a randomized clinical trial. Diabetes Educ.

[13] Melko CN, Terry PE, Camp K, Min X, Healey ML (2009). Diabetes health coaching improves medication adherence: A pilot study. American Journal of Lifestyle Medicine.

[14] Cuspidi C, Meani S, Fusi V, Salerno M, Valerio C, Severgnini B, Catini E, Leonetti G, Magrini F, Zanchetti A (2004). Home blood pressure measurement and its relationship with blood pressure control in a large selected hypertensive population. J Hum Hypertens.

[15] Logan AG, McIsaac WJ, Tisler A, Irvine MJ, Saunders A, Dunai A, Rizo CA, Feig DS, Hamill M, Trudel M, Cafazzo JA (2007). Mobile phone-based remote patient monitoring system for management of hypertension in diabetic patients. Am J Hypertens.

[16] Halifax NV, Cafazzo JA, Irvine MJ, Hamill M, Rizo CA, McIssac WJ, Rossos PG, Logan AG (2007). Telemanagement of hypertension: a qualitative assessment of patient and physician preferences. Can J Cardiol.

[17] Nguyen HQ, Wolpin S, Chiang K-C, Cuenco D, Carrieri-Kohlman V (2006). Exercise and symptom monitoring with a mobile device. AMIA Annu Symp Proc.

[18] Wang DH, Kogashiwa M, Ohta S, Kira S (2002). Validity and reliability of a dietary assessment method: the application of a digital camera with a mobile phone card attachment. J Nutr Sci Vitaminol (Tokyo).

[19] Arsand E, Tufano JT, Ralston JD, Hjortdahl P (2008). Designing mobile dietary management support technologies for people with diabetes. J Telemed Telecare.

[20] Lester RT, Mills EJ, Kariri A, Ritvo P, Chung M, Jack W, Habyarimana J, Karanja S, Barasa S, Nguti R, Estambale B, Ngugi E, Ball TB, Thabane L, Kimani J, Gelmon L, Ackers M, Plummer FA (2009). The HAART cell phone adherence trial (WelTel Kenya1): a randomized controlled trial protocol. Trials.

[21] Abebe NA, Capozza KL, Des Jardins TR, Kulick DA, Rein AL, Schachter AA, Turske SA (2013). Considerations for community-based mHealth initiatives: insights from three Beacon Communities. J Med Internet Res.

[22] Free C, Phillips G, Galli L, Watson L, Felix L, Edwards P, Patel V, Haines A (2013). The effectiveness of mobile-health technology-based health behaviour change or disease management interventions for health care consumers: a systematic review. PLoS Med.

[23] Olah ME, Gaisano G, Hwang SW (2013). The effect of socioeconomic status on access to primary care: an audit study. CMAJ.

[24] Chen E, Miller GE (2013). Socioeconomic status and health: mediating and moderating factors. Annu Rev Clin Psychol.

[25] Prus SG (2011). Comparing social determinants of self-rated health across the United States and Canada. Soc Sci Med.

[26] (2013). Statistics Canada.

[27] Creese G, Wiebe B (2012). ‘Survival Employment’: Gender and deskilling among African immigrants in Canada. International Migration.

[28] Quinn CC, Clough SS, Minor JM, Lender D, Okafor MC, Gruber-Baldini A (2008). WellDoc mobile diabetes management randomized controlled trial: change in clinical and behavioral outcomes and patient and physician satisfaction. Diabetes Technol Ther.

[29] Quinn CC, Shardell MD, Terrin ML, Barr EA, Ballew SH, Gruber-Baldini AL (2011). Cluster-randomized trial of a mobile phone personalized behavioral intervention for blood glucose control. Diabetes Care.

[30] Quinn CC, Gruber-Baldini AL, Shardell M, Weed K, Clough SS, Peeples M, Terrin M, Bronich-Hall L, Barr E, Lender D (2009). Mobile diabetes intervention study: testing a personalized treatment/behavioral communication intervention for blood glucose control. Contemp Clin Trials.

[31] Levine JA (2011). Poverty and obesity in the U.S. Diabetes.

